# Full-Spectrum Analysis of Bioactive Compounds in Rosemary (*Rosmarinus officinalis* L.) as Influenced by Different Extraction Methods

**DOI:** 10.3390/molecules25204599

**Published:** 2020-10-09

**Authors:** Yashaswini Sharma, Ravikishore Velamuri, John Fagan, Jim Schaefer

**Affiliations:** 1 Department of Sustainable Living, Maharishi International University, Fairfield, IA 52557, USA; 2 Health Research Institute, Fairfield, IA 52556, USA; kishore@hrilabs.org; 3 Health Research Institute & College of Sustainable Living, Maharishi International University, Fairfield, IA 52556, USA; john.fagan@HRILabs.org; 4 Soil Technologies Corp., Fairfield, IA 52556, USA; jsstc108@yahoo.com

**Keywords:** rosemary, rosmarinic acid, ursolic acid, Soxhlet extraction, sonication, UHPLC-ESI-QTOF-MS

## Abstract

*Rosmarinus officinalis* is a potent antioxidant herb rich in polyphenols. Ultra-high-performance liquid chromatography, coupled with electrospray ionization and quadrupole-time of flight mass spectrometry (UHPLC-ESI-QTOF-MS), enables an exhaustive, full-spectrum analysis of the molecular constituents of natural products. The study aimed to develop a rapid UHPLC method to contribute new insights into the phytochemical composition of rosemary and to assess the performance of nine different procedures for extraction. These include fresh tissue homogenization, fresh and dry leaf decoction, and their respective fermentation, Soxhlet extraction, and sonication using water and methanol. Different extraction methods were found to recover quite different groups of polyphenols within 11 min during 20 min of analysis. Soxhlet extraction, yielded very high concentrations of rosmarinic acid (33,491.33 ± 86.29 µg/g), luteolin-7-*O*-glucoside (209.95 ± 8.78 µg/g), carnosic acid (2915.40 ± 33.23 µg/g), carnosol (22,000.67 ± 77.39 µg/g), and ursolic acid (5144.27 ± 28.68 µg/g). UHPLC-ESI-QTOF-MS enabled the detection of more than 50 polyphenols, including phenolic acids, flavonoids, and terpenoids in the various extracts. Of these, sagerinic acid ([M − H]^−^ *m/z* 719.16), salvianolic acid A ([M − H]^−^ *m/z* 493.11) and B ([M − H]^−^ *m/z* 717.15), and a pentacyclic triterpenoid corosolic acid ([M − H]^−^ *m/z* 471.34) were detected for the first time in rosemary. Soxhlet extraction was found to be the most efficient method, followed by dry leaf decoction. The UHPLC-ESI-QTOF-MS methodology for the analysis proved to be very efficient in the identification and characterization of targeted and untargeted bioactive molecules in the rosemary.

## 1. Introduction

Rosemary (*Rosmarinus officinalis* L.) is an evergreen perennial culinary herb belonging to the family Lamiaceae and is popularly used as a spice and medicine. The herb is traditionally used to treat memory-related disorders, hypertension, headache, insomnia, and diseases related to the respiratory system [1,2]. Rosemary is considered as a powerful cardiac stimulant, a strong antiseptic, antispasmodic, carminative, emmenagogue, and nervine tonic, and is used to cure arthritis, dandruff, and skin diseases [3,4]. The essential oil from its leaves is used as a natural antimicrobial, pesticide, and insect repellent [5]. The therapeutic properties of rosemary have been attributed to its phytochemical constituents, such as phenolic acids, flavonoids, and terpenoids [6,7].

Ultra-high-performance liquid chromatography and electrospray ionization, coupled with quadrupole-time of flight mass spectrometry (UHPLC-ESI-QTOF-MS), is improved technology for separation and investigation of complex polyphenols in food samples [8]. UHPLC provides rapid, high-resolution, along with higher selectivity and sensitivity, while ESI-QTOF-MS identifies multiple targeted and untargeted constituents of the sample in real-time. Characterization of unknown compounds in UHPLC is based on their exact mass (*m/z*) and *m/z* fragmentation pattern with high *m/z* resolution; further, this technology could also be used to distinguish isobaric compounds by exact mass with different elemental positions [8,9]. Hence, the study was conducted to develop a rapid analytical methodology to provide new insights into the range of phytochemicals present in rosemary and the relative amounts of these compounds. 

There are only a few studies reported on the phytochemical profiling of rosemary, and these mainly describe alcohol-based extraction. So far, minimal effort has been made to evaluate the quality of the herb and extracts hereof, based on traditional and industrial methods. There is some evidence in Ayurvedic classics that fermentation enhances the therapeutic and biochemical properties of herbal drugs [10,11,12]. At the same time, ultrasound extraction for a short period (1–2 h) at low frequencies (40 kHz) is reported to increase the yield of alkaloids in herbal extracts and to significantly reduce extraction time and solvent consumption, resulting in comparable or superior extracts to those obtained using decoction and maceration [13,14]. Hence, in the present investigation, an effort has been made to assess several extraction methods in terms of efficiency and final concentrations of critical bioactive constituents of rosemary. The extraction methods evaluated included aqueous extraction, decoction, Soxhlet’s extraction, Ayurvedic fermentation, and sonic extractions in rosemary. 

## 2. Results and Discussion

### 2.1. Quantification of Bioactive Compounds by UHPLC-ESI-QTOF-MS 

The concentrations of different bioactive compounds, caffeic acid, rosmarinic acid, luteolin-7-*O* glucoside, carnosic acid, ursolic acid, and carnosol (µg/g) analyzed through UHPLC-ESI-QTOF-MS as influenced by different extraction methods are presented in Table 1. Among all the extractions, Soxhlet extract (T_7_) recorded significantly higher rosmarinic acid (33,491.33 ± 86.29 µg/g), luteolin-7-*O*-glucoside (209.95 ± 8.78 µg/g), carnosic acid (2915.40 ± 33.23 µg/g), carnosol (22,000.67 ± 77.39 µg/g), and ursolic acid (5144.27 ± 28.68 µg/g). Soxhlet extraction combined with methanol solvent might enhance the solubility of polyphenols, flavonoids, and other bioactive compounds present in herbs, maximizing the extraction of phytochemical constituents [15]. All the extractions in the study yielded considerable amounts of rosmarinic acid ranging from 0.26 µg/g to 33.49 mg/g, contributing substantially to the high antioxidant potential of the extracts. The results are in good agreement with previous studies, in which rosmarinic acid concentrations were reported in the range of 5.6 µg–2.34 mg/g in rosemary leaf extracts from Serbia and Iraq [16,17]; rather, various extraction procedures of our study in rosemary (T_4_–T_7_, T_9_) recorded higher rosmarinic acid concentrations than the previous reports.

The decoction from dry leaf powder (T_5_) recorded significantly higher levels of caffeic acid (322.02 ± 3.39 µg/g) as compared to other treatments. Fresh leaf decoction also contained a considerable amount of rosmarinic acid, carnosic acid, and carnosol. Carrying out the decoction process using water helps to dissolve the maximum amounts of these water-soluble compounds [18]. Levels of polyphenols and terpenoid compounds were significantly higher in dry leaf decoction compared to fresh leaf decoction, primarily because the amount of biomass that could be extracted was immense. The conversion rate of fresh to dry rosemary was 33%. Among the traditional extraction methods, dry leaf decoction (T_5_) and its fermentation (T_6_) were found to yield higher levels of caffeic acid and rosmarinic acid. Fermentation significantly enhanced the rosmarinic acid levels in both T_4_ and T_6_. Fermentation also enhanced caffeic acid content in both fermented fresh homogenized tissue extract (T_3_) and fresh leaf decoction (T_4_). This may be due to the microbial transformation of chemical compounds and better extraction of herbal constituents due to the production of alcohol during fermentation. It may also be the case that extraction was facilitated by fermentation due to the release of bacterial enzymes that broke down cell walls of the rosemary plant, making compounds more accessible to extraction by a solvent [19,20]. In the present study, fermentation enhanced the phenolic acids; however, it reduced flavonoid content, luteolin-7-glucoside and diterpenoids, carnosic acid, and carnosol. It is likely that the oxidation of phenolic compounds during fermentation reduced the levels of certain polyphenols. Similar results were reported in *Centella asiatica* and *Orthosiphon aristatus* [21,22,23].

Ultrasound extraction using methanol (T_9_) resulted in significantly higher concentrations of rosmarinic acid and ursolic acid as compared to other fresh extraction and fermentation procedures. Ultrasound is known to disrupt plant cell walls, thereby facilitating the release of extractable compounds and enhancing mass transport of solvent from plant cells into the solvent phase. This effect boosts compound recovery, mostly when an optimal solvent, in this case, methanol, was used [13,24]. In contrast to sonication with methanol, sonication with water yielded the lowest levels of phenolic acids and flavonoids of all extraction methods employed. This is not surprising since the complex structures of phenolic compounds cause them to be rather insoluble in aqueous media [25]. Among aqueous and methanol extraction, methanol extracts showed significantly higher polyphenols and terpenoids, especially in Soxhlet and sonic extracts. This might be due to the higher solubility of complex bioactive compounds in organic solvents than the aqueous base [25,26]. The herb was found to contain a considerable quantity of rosmarinic acid and ursolic acid in most of the extractions, responsible for its healing properties, supporting traditional usage for treating gastrointestinal inflammation, colitis, colon cancer, and nervous system inflammation [27].

### 2.2. Identification and Characterization of Bioactive Constituents in R. officinalis 

**Figure 1:**
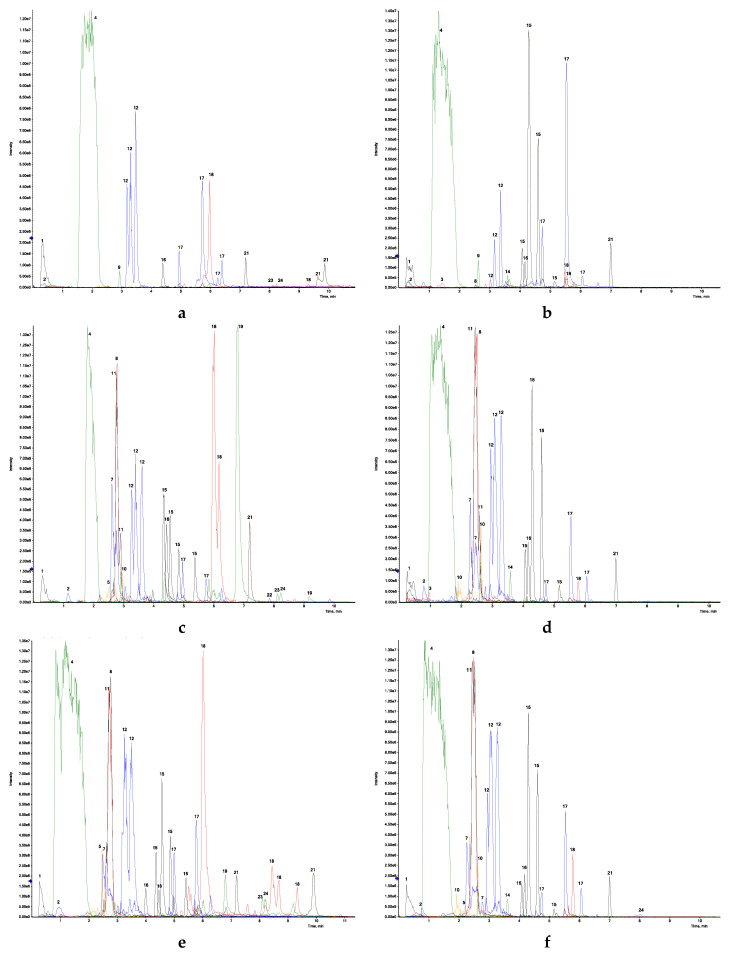

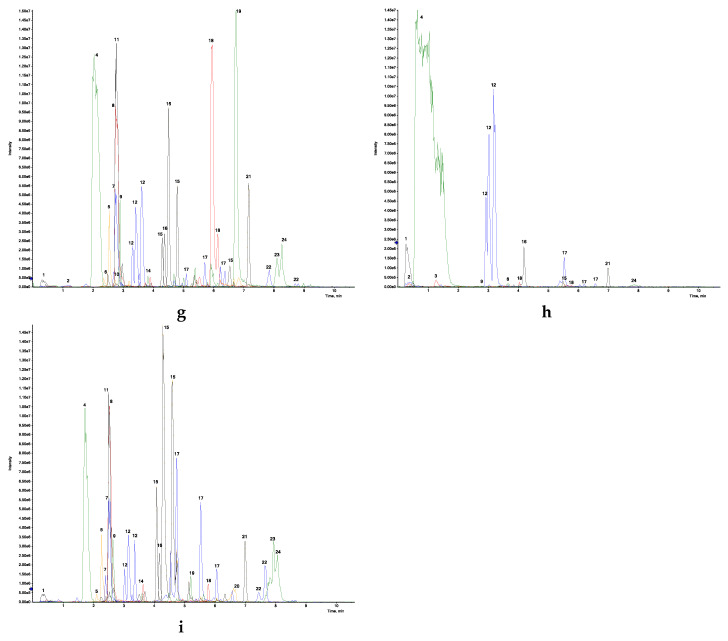
Chromatogram representing relative abundance of polyphenols and terpenoids in different extracts of rosemary leaves (T_1_–T_9_) analyzed through ultra-high-performance liquid chromatography with electrospray ionization and quadrupole-time of flight mass spectrometry (UHPLC-ESI-QTOF-MS) (intensity versus elution time); (**a**) Fresh tissue homogenization (T_1_); (**b**) Fresh homogenized tissue extract fermentation (T_3_); (**c**) Fresh leaf decoction (T_2_); (**d**) Fresh leaf decoction fermentation (T_4_); (**e**) Dry leaf decoction (T_5_); (**f**) Dry leaf decoction fermentation (T_6_); (**g**) Soxhlet extraction (T_7_); (**h**) Sonication with water (T_8_); (**i**) Sonication with methanol (T_9_). Peak numbers refer to: 1—quinic acid; 2—caffeic acid; 3—coumaric acid; 4—gallocatechin; 5—rosmarinic acid-3-*O*-glucoside; 6—luteolin-7-*O*-glucoside; 7—salvianolic acid B; 8—rosmarinic acid; 9—hesperidin; 10—Salvianolic acid A; 11—Sagerinic acid; 12—Luteolin 3’-acetyl-*O*-glucuronide; 13—Apigenin; 14—Diosmetin; 15—Rosmanol; 16—Pectolinarigenin; 17—Rosmadial; 18—Carnosol; 19—Carnosic acid; 20—Corosolic acid; 21—12-methoxy-carnosic acid; 22—Micromeric acid; 23—Betulinic acid; 24—Ursolic acid.

Rapid separation polyphenol molecules were achieved within the first 11 min of 20 min of UHPLC analysis duration. More than 50 polyphenolic compounds have been identified by UHPLC-ESI-QTOF-MS under negative electrospray ionization conditions [M − H]^−^ based on their retention times, molecular weights, and mass (*m/z*) fragmentation patterns. The study was focused on negative ionization mode [M − H]^−^ because it is reported to be more sensitive for analysis of phenolic acids and flavonoids, compared to positive ionization mode [28,29,30]. The phenolic compounds in rosemary extracts were mostly flavonoids, phenolic acids, and terpenoids. The terpenoids included diterpenoids largely, along with a few triterpenoids. The data are presented in three groups: polyphenols in homogenous aqueous extraction (T_1_) and its fermentation (T_3_) (Table 2); fresh and dry leaf decoctions (T_2_ and T_5_), and their respective fermentations (T_4_ and T_6_) (Table 3); industrial extractions Soxhlet (T_7_) and sonication with water and methanol (T_8_ and T_9_) (Table 4). Chromatograms depicting the intensity of polyphenols in different rosemary extracts (T_1_–T_9_) versus retention time (min) are presented in Figure 1a–i. The compounds without reference standards were identified tentatively by comparing the mass spectra data, ion fragmentation, and molecular weight (*m/z*) with data available in the literature [17,31] and the mass spectral library obtained from the National Institutes of Standards and Technology (NIST-2017), AOI (All-in-One) spectral library from Sciex, MoNA (MassBank of North America), and HILIC (Hydrophilic Interaction Liquid Chromatography) library database from University of California, Davis.

As shown in Table 2, there were about 41 polyphenols detected in fresh homogenized tissue extraction (T_1_) and its fermentation (T_3_); among them, T_1_ contained 30 and T_3_ contained 33 polyphenols. The chromatogram in Figure 1a,b represents the relative intensity of phenolic compounds in T_1_ and T_3_, respectively. The fermented sample (T_3_) was found to have a higher intensity of rosmanol and rosmadial compared to T_1_, whereas the relative intensities of luteolin 3-acetyl-*O*-glucuronide and carnosol were high in T_1_. Rosemary leaf decoctions (fresh and dry, T_2,_ and T_5_, respectively) were found to be a more efficient extraction method for polyphenol content. In total, 54 phenolic compounds were identified in T_2_ and T_5_ and their fermented extracts (T_4_ and T_6_) (Table 3). The intensities of phenolic compounds and terpenoids were lower in the fermented decoctions compared to the fresh and dry leaf decoctions (Figure 1c–f). This is consistent with earlier reports with other herbs, indicating that prolonged fermentation can break down phenolic compounds resulting in decreased antioxidant potential [22].

A large group of phenolic compounds was observed in Soxhlet and sonicated methanol extracts (T_7_ and T_9_, respectively). Of the 59 polyphenols, 11 were tentatively identified as phenolic acids and seventeen as terpenoids (Table 4). In methanolic samples, the intensity of terpenoid compounds, rosmanol, rosmadial, carnosol, carnosic acid, and ursolic acid, was found to be very high, as is depicted in their chromatograms in Figure 1g,i. Sonication of rosemary in water (T_8_) resulted in a much lower number of polyphenols, compared to methanolic extraction (Figure 1h). This is likely due to the lower solubility of complex terpenoids and phenolic molecules in water compared to methanol [24]. Out of 11 identified phenolic compounds in methanol extracts, quinic acid, syringic acid, chlorogenic acid, caffeic acid, 4-*O*-caffeoylquinic acid, p-coumaric acid, and rosmarinic acid have been reported before [16,30]. However, isoferulic acid ([M − H]^−^ *m/z* 193.05), sagerinic acid ([M − H]^−^ *m/z* 719.16), and salvianolic acid A ([M − H]^−^ *m/z* 493.11) and B ([M − H]^−^ *m/z* 717.15) were reported herein for the first time in rosemary extracts, based on comparison of the *m/z* ion fragmentation pattern of the observed compounds compared to those in the NIST MS library. Sagerinic acid was found in very high intensities (T_2_, T_4_, T_5_, T_6_, T_7_, and T_9_—Figure 1), and it shared some (*m/z*) MS/MS ion fragments (359.08) with rosmarinic acid ([M − H]^−^ *m/z* 359.08). Lu and Foo reported sagerinic acid as possible derivatives of rosmarinic acid, since they are structurally related [32]. Similarly, syringic acid ([M − H]^−^ at *m/z* 197.05), 4-*O*-caffeoylquinic acid ([M − H]^−^ at *m/z* 353.09), rosmarinic acid ([M − H]^−^ at *m/z* 359.08), and methyl rosmarinate ([M − H]^−^ at *m/z* 373.09) all shared many of the same MS/MS (*m/z*) ion fragments (179.03), since they were found to be dimers of caffeic acid.

A large group of flavonoids has been reported in this study, and most of them were derivatives of luteolin ([M − H]^−^ at *m/z* 285.04), hesperidin ([M − H]^−^ at *m/z* 609.18), and apigenin ([M − H]^−^ at *m/z* 269.04). Similar results were obtained from LC/MS analysis of rosemary herb from the USA and Iraq [17,31]. Very high intensities of gallocatechin ([M − H]^−^ at *m/z* 305.07) were observed in all rosemary extracts (Figure 1a–i), and this was reported in previous studies [6,17]. Gallocatechin is a flavan-3-ol found predominantly in fruit peels, and gallocatechin was reported to be responsible for the high antioxidant potential of the herb [33,34,35]. In the present study, some flavonoid compounds have been detected for the first time in rosemary, viz., phlorizin ([M − H]^−^ at *m/z* 435.13) in Soxhlet extract (Table 4) and pectolinarigenin ([M − H]^−^ at *m/z* 313.07) in all the extracts (T_1_–T_9_). Phlorizin was earlier found in tree barks of the Rosaceae family, and the studies indicated high antidiabetic property of the drug [36]. Pectolinarigenin was also reported before in rosemary as dimethoxyflavone with similar fragment ions, and it was found to have potent anti-inflammatory and anticancer properties [31,37,38]. Further, these newly detected flavonoids and phenolic acids can be confirmed by procuring respective standards or by using advanced techniques like nuclear magnetic resonance (NMR) spectroscopy for identification and confirmation of unknown molecules. The presence of three peaks for luteolin 3’-acetyl-*O*-glucuronide ([M − H]^−^ at *m/z* 503.08) eluted at 3.29, 3.38, and 3.62 min with similar *m/z* fragments (443.06, 245.47) could be observed in chromatograms of all extracts (T_1_–T_9_). Previously, multiple peaks for luteolin 3’-acetyl-*O*-glucuronide in rosemary extract were reported by Borras-Linares [17]. These are probably due to the existence of multiple positional isomers of this compound in rosemary.

There were about 17 terpenoid compounds that have been tentatively identified in methanolic extracts of rosemary, out of which 12 were diterpenoids (Table 4). Rosmanol ([M − H]^−^ at *m/z* 345.17), rosmadial ([M − H]^−^ at *m/z* 343.15), carnosol ([M − H]^−^ at *m/z* 329.18), carnosic acid ([M − H]^−^ at *m/z* 331.19), and 12-methoxy carnosic acids ([M − H]^−^ at *m/z* 345.21) were the major diterpenoids present in higher intensities in T_2_, T_3_, T_4_, T_5_, T_6_, T_7_, and T_9_ (Figure 1). The presence of more than one peak corresponding to the same molecular mass but different elution times was due to the presence of isomers, especially in rosmanol and rosmadial. Rosmanol ([M − H]^−^ *m/z* 345.17) eluted at four different retention times, with the same ion fragmentation (MS^2^ *m/z* fragments 301.1779, 183.1668). Rosmadial ([M − H]^−^ *m/z* 343.15) and its isomers also resulted in three to four peaks with similar fragmentation patterns (MS^2^ *m/z* 299.16). Similar peaks were obtained in rosmadial of sage and rosemary extracts during the chromatographic determination of polyphenols [31,39]. A diterpenoid, triptolidenol ([M − H]^−^ at *m/z* 375.15), was detected only in T_9_. Five pentacyclic triterpenoid compounds viz., asiatic acid ([M − H]^−^ at *m/z* 487.33), corosolic acid ([M − H]^−^ at *m/z* 471.34), micromeric acid ([M − H]^−^ at *m/z* 453.34), betulinic acid, and ursolic acid ([M − H]^−^ at *m/z* 455.35) were tentatively detected in methanolic samples (Table 4). Previously, betulinic acid, ursolic acid, and micromeric acids were determined in rosemary leaves [17,31]. Betulinic acid in the herbs was found to have potent antiviral activity against severe acute respiratory syndrome coronavirus [40]. A triterpenoid corosolic acid was tentatively identified for the first time in the rosemary extract of T_7_. Asiatic acid was present in T_2_ and T_7_; micromeric acid and betulinic acid were detected in T_2_, T_5_, T_7_, and T_9_; ursolic acid was found in all extracts. Pentacyclic triterpenoids reported having several medicinal properties, especially anti-inflammatory, anticancer, and antidiabetic potential [41,42]. Even though ursolic acid and betulinic acid have the same pseudomolecular weight ([M − H]^−^ at *m/z* 455.35), the former was identified through the reference standard, and the later molecule was confirmed by comparison to the NIST mass spectral library. Besides, several other compounds were detected in significant amounts in certain extracts that were not represented in the mass spectral databases available. 

Even though comparison of high resolution, accurate mass, LC-MS/MS chromatograms and m/z fragmentation patterns of observed compounds with high-resolution mass spectral libraries is a very effective approach for the identification and characterization of known and previously unknown compounds, this approach is limited to those compounds represented in MS/MS libraries. Our analyses generated mass spectral data for a large number of yet-to-be-identified phenolic compounds present in the rosemary extracts, which we analyzed. As mass spectral libraries expand, the data that we have already gathered can be further analyzed to structurally identify additional polyphenols based on *m/z* fragmentation patterns. In addition, if further inspection of our data identifies unnamed compounds that are of particular interest, possibly due to a high abundance of other features of interest, then additional work can be done to isolate and identify those compounds using nuclear magnetic resonance (NMR) spectroscopy and other approaches.

## 3. Materials and Methods

### 3.1. Herb Collection

Rosemary (*Rosmarinus officinalis* L.) leaves after eight months of planting were harvested from the Regenerative Organic Farm, Maharishi University of Management, Fairfield, Iowa. Freshly harvested leaves were used for fresh extractions, whereas air-dried leaf powder was used for dry extractions. The sample was submitted to Ada Hayden Herbarium (ISC/IA), Iowa State University, Iowa, USA, and obtained the accession no. ISC-454695.

### 3.2. Chemicals

LCMS grade acetonitrile and methanol were purchased from Honeywell, Burdick, and Jackson, USA. LCMS grade formic acid and glacial acetic acid were procured from Merck, Germany. Caffeic acid, rosmarinic acid, carnosic acid, ursolic acid, and luteolin 7-glucoside were purchased from Toronto Research Chemicals, Canada. Carnosol and ^13^C- caffeic acid were purchased from Cayman Chemical, USA. Ultrapure water from the Milli-Q, A10 water purification system (Millipore Sigma, Madison, WI, USA) was used throughout the experiment. 

### 3.3. Preparation of R. Officinalis Extracts 

There were nine different sample extraction methods used as treatments for liquid chromatographic analysis.

T_1_: Fresh aqueous extraction by tissue homogenization—10 g of fresh leaf samples was macerated in 100 mL Milli-Q water at room temperature and fresh leaf juice was extracted by filtering through cellulose filter paper. 

T_2_: Fresh leaf decoction—10 g of fresh leaves was chopped into 1–2 cm pieces and boiled in 200 mL Milli-Q water at 100–110 °C temperature until the volume was reduced to 100 mL. The extract was cooled, filtered, and used for the analysis. 

T_3_: Fresh tissue homogenized extract fermentation—homogenized fresh leaf tissue extract (T_1_) was fermented by adding 24% sugar and 10 mg of the activated wine yeast *Saccharomyces cerevisiae* for 60 days; the resultant clear fermented extract was filtered and used for analysis. 

Preparation of yeast (*Saccharomyces cerevisiae*) inoculum: 10 mg of commercial wine yeast culture (Lalvin EC-1118 strain—produced in Canada from grape skin) was dissolved in 2 mL of warm water (43 °C) for 10 min; as the yeast activates at warm water, it starts producing small bubbles. A total of 2 mL of such activated *Saccharomyces cerevisiae* culture was added into the rosemary extracts for fermentation.

T_4_: Fresh leaf decoction fermentation—T_2_ samples were fermented by adding 24% sugar and the activated wine yeast *Saccharomyces cerevisiae* culture for 60 days.

T_5_: Dry leaf decoction—10 g of leaf powder was boiled in 200 mL Milli-Q water at 100–110 °C temperature until the volume was reduced to 100 mL, and the extract was cooled, filtered, and used for further analysis.

T_6_: Dry leaf decoction fermentation—T_5_ samples were fermented by adding 24% sugar and the activated wine yeast *Saccharomyces cerevisiae* culture for 60 days.

T_7_: Soxhlet extraction—10 g of leaf powder was extracted using 250 mL LCMS grade methanol in the Soxhlet apparatus at 70 °C for 6 h, and the volume was further reduced to 100 mL by a vacuum evaporator and filtered through a 0.2 µ Nalgene filter unit from Thermo Fisher Scientific Inc. (Waltham, MA, USA).

T_8_ and T_9_: Sonic/ultrasound extraction in water and methanol, respectively—10 g of leaf powder was extracted in 100 mL Milli-Q water and methanol (50 °C) for 2 h with a frequency of 40 kHz in a Bransonic-52 ultrasonic bath unit from Branson, USA. 

All extractions were made in triplicates and stored protected from light at −20 °C until chromatographic analysis.

### 3.4. UHPLC-ESI-QTOF-MS Method Development

*R. officinalis* samples were analyzed by ultra-high-performance liquid chromatography, electrospray ionization coupled with quadrupole-time of flight mass spectrometry (UHPLC-ESI-QTOF-MS). The analysis was carried out by reverse-phase UHPLC (Shimadzu Nexera, Kyoto, Japan) directly connected to a quadrupole Time-of-Flight (QTOF) Triple TOF 5600 mass spectrometer (AB SCIEX, Concord, ON, Canada). The autosampler (Shimadzu SIL30AC, Kyoto, Japan) was operated in direct injection mode, filling a 50 µL loop with 10 µL analyte for optimal sample delivery reproducibility. Samples were passed through the C_18_ column (Kinetex XB, 1mm I.D. × 5 cm, 2.6 µm, particle size, 100 Å) and eluted at a flow rate of 250 µL/min. Pumps (Shimadzu LC30AD, Kyoto, Japan) were operated in the following multi-step linear gradient with different proportion of mobile phase B: 0 min, 10% B; 10 min, 90% B; 12.5 min, 90% B; 15 min, 10% B; 20 min, 10% B, with a total runtime of 20 min including mobile phase equilibration. Mobile phases A and B used were 0.1% of acetic acid made in Milli-Q water and acetonitrile, respectively. The column oven (Shimadzu CTO30A, Kyoto, Japan) was set to 40 °C.

### 3.5. Identification and Quantification of Polyphenols

Mass spectra and tandem mass spectra data were recorded in electrospray ionization (ESI), “negative-ion” mode with a resolution of ~35,000 full-width half-maximum on the QTOF 5600. The ion spray needle voltage was at −4500 V with drying gas temperature 600 °C, and ion source Gas 1 (nebulizer) and Gas 2 (heater) values were 50 psi each. The collision-energy values for QTOF MS were at 5 eV and for MS/MS experiments at 25 eV with a spread of 15 eV. For collision-induced dissociation tandem mass spectrometry, the mass window for precursor ion selection of the quadrupole mass analyzer was set to ±1 *m/z*. The precursor ions were fragmented in a collision cell using nitrogen as the collision gas. Data independent acquisitions (DIA) with SWATH-MS^2^ cover the mass range of *m/z* 50–1000 in 16 segments (15 × 48.5 ms), yielding a cycle time of 0.8268 s, which includes one 50 msec MS^1^ scan. During the execution of the liquid chromatography method, the mass spectrometer was externally calibrated using a known mixture of masses from Sciex (P/N 4460134, AB SCIEX, Concord, ON, Canada). 

Quantitative analysis was performed by diluting the extracted samples with 0.1% formic acid (1/10 to 1/10,000) in order to quantify the samples within the linearity range of the standard calibration curve, avoiding MS signal saturation. The method was validated for sensitivity and precision. The standard calibration curves were constructed for quantification of caffeic acid, rosmarinic acid, luteolin-7-*O*-glucoside, carnosol, carnosic acid, and ursolic acid. Table 5 represents calibration parameters, including limits of quantification (LOQ), calibration range, equations, and slope. All samples were extracted and analyzed in triplicate. Unknown polyphenolic compounds and flavonoids were identified based on their accurate mass (*m/z*) and molecular (*m/z*) ion fragmentation patterns using Peak view Software (ver.2.2, AB SCIEX, Concord, ON, Canada), Master view, Library view (AB SCIEX, Concord, Canada), National Institute of Standards and Technology (NIST), and the AOI database. 

### 3.6. Statistical Analysis

The results of polyphenol quantification were expressed as mean ± SD. The data were analyzed statistically by using single-factor ANOVA in MS Excel software. The critical difference at 1% level of significance or Tukey’s HSD (Honestly Significant Difference) test (at *p* < 0.01) was used to compare the significant difference between the treatments [43].

## 4. Conclusions

Rapid separation of most of the polyphenols was achieved within the first 11 min during 20 min of UHPLC analysis. Among all the extraction methods, Soxhlet extraction yielded significantly higher levels of polyphenols, both in terms of numbers of compounds and levels of these compounds. Dry leaf decoction was found to be the next best extraction method for rosemary, yielding significantly higher caffeic acid, rosmarinic acid, carnosol, carnosic acid, and flavonoids. This might be the best method for large-scale commercial extraction. Sonic extraction with methanol was found to be the second-best for the extraction of rosmarinic acid and ursolic acid. Most of the extractions in the study yielded a high concentration of rosmarinic acid up to 33.49 mg/g, contributing substantially to the high antioxidant potential of the extracts. As compared to previous studies, the rosemary extract of our study recorded a higher concentration of bioactive constituents, indicating the quality of the herb grown in Fairfield, Iowa, USA. The present study also helps to choose an efficient extraction method for obtaining maximum polyphenolic and terpenoid content, not only in rosemary but also in similar herb species. UHPLC-ESI-QTOF-MS methodology for the analysis proved to be very efficient in the identification and characterization of targeted and untargeted phenolic compounds present in the rosemary. However, there is substantial scope to investigate structurally and functionally the many potentially interesting but yet-unidentified phenolic compounds present in rosemary. 

## Figures and Tables

**Table 1:** Polyphenol and terpenoid content (µg/g) in different extraction of Rosemary analyzed by ultra-high-performance liquid chromatography and electrospray ionization, coupled with quadrupole-time of flight mass spectrometry (UHPLC-ESI-QTOF-MS). (Figures S1–S6)

Treatment	Polyphenol and Terpenoid Content (µg/g) in Rosemary					
	Caffeic Acid	Rosmarinic Acid	Luteolon-7-*O*-Glucoside	Carnosic Acid	Carnosol	Ursolic Acid
T_1_	6.02 ± 0.08 ^b^	1.51 ± 0.07 ^a^	1.59 ± 0.22 ^a^	0.64 ± 0.01 ^a^	112.06 ± 0.61 ^b^	5.32 ± 0.17 ^a^
T_2_	12.30 ± 0.33 ^c^	1124.03 ± 13.62 ^b^	7.14 ± 0.14 ^bc^	1374.63 ± 7.72 ^b^	171.52 ± 1.59 ^b^	6.08 ± 0.17 ^a^
T_3_	13.03 ± 0.70 ^c^	2.51 ± 0.35 ^a^	ND	0.25 ± 0.01 ^a^	0.54 ± 0.01 ^a^	0.36 ± 0.01 ^a^
T_4_	38.56 ± 1.58 ^e^	5428.47 ± 19.69 ^c^	ND	0.97 ± 0.01 ^a^	1.91 ± 0.04 ^a^	2.36 ± 0.07 ^a^
T_5_	322.02 ± 3.39 ^g^	13,310.13 ± 26.12 ^d^	130.53 ± 5.41 ^d^	2671.83 ± 20.03 ^c^	417.21 ± 1.99 ^c^	89.20 ± 1.92 ^b^
T_6_	106.83 ± 1.49 ^f^	15,242.40 ± 43.62 ^e^	4.67 ± 0.68 ^ab^	5.39 ± 0.48 ^a^	10.82 ± 0.59 ^a^	6.09 ± 0.19 ^a^
T_7_	40.55 ± 0.03 ^e^	33,491.33 ± 86.29 ^g^	209.95 ± 8.78 ^e^	2915.40 ± 33.23 ^d^	22,000.67 ± 77.39 ^d^	5144.27 ± 28.68 ^d^
T_8_	2.40 ± 0.06 ^a^	0.26 ± 0.00 ^a^	0.97 ± 0.01 ^a^	ND	2.19 ± 0.19 ^a^	10.37 ± 0.88 ^a^
T_9_	23.77 ± 1.63 ^d^	15,944.00 ± 36.39 ^f^	9.11 ± 0.35 ^c^	6.97 ± 0.34 ^a^	34.98 ± 1.10 ^a^	1042.88 ± 11.33 ^c^
**Mean**	62.83	9393.85	52.99	872.01	2527.99	700.77
**F Test**	**	**	**	**	**	**
**SEM ±**	0.85	21.02	0.81	7.61	14.90	5.95
**CD at 1** **%**	3.46	85.56	3.28	31.00	60.67	24.21

** Significant at 1% level, values followed by different letters indicate a significant difference between the treatments at *p* < 0.01; ND—Not detected. Treatment Details: T_1_: Fresh tissue homogenization; T_2_: Fresh leaf decoction; T_3_: Fresh homogenized tissue extract fermentation; T_4_: Fresh leaf decoction fermentation; T_5_: Dry leaf decoction; T_6_: Dry leaf decoction fermentation; T_7_: Soxhlet extraction; T_8_: Sonic extraction—aqueous; T_9_: Sonic extraction—methanol.

**Table 2:** Bioactive compounds in fresh tissue homogenization (T_1_) and its fermentation (T_3_) of *Rosmarinus officinalis* identified by UHPLC-ESI-QTOF-MS.

Sl No	Compound	RT (min)	Mass [M − H]^−^ (*m/z*)	Formula	Fragments	T_1_	T_3_
1.	Quinic acid	0.42	191.05681	C_7_H_12_O_6_	85.0297 (42) *, 127.0401 (24), 59.0165 (13)	+	+
2.	Caffeic acid	0.78	179.03520	C_9_H_8_O_4_	135.0438 (100), 134.0370 (21)	+	+
3.	*p*-Coumaric acid	1.44	163.04014	C_9_H_8_O_3_	119.0500 (100)	+	+
4.	Gallocatechin	1.65	305.07057	C_15_H_14_O_7_	225.1126 (69), 96.9597 (16), 98.9574 (8)	+	+
5.	Luteolin 7*-O*-rutinoside	2.18	593.15382	C_27_H_30_O_15_	297.0740 (8), 285.0410 (6)	+	−
6.	Salvianolic acid B	2.29	717.14274	C_36_H_30_O_16_	519.0900 (62), 339.0494 (33)	+	−
7.	Rosmarinic acid	2.58	359.07906	C_18_H_16_O_8_	161.0236 (100), 197.0449 (72), 179.0347 (68), 135.0448 (7)	+	+
8.	Isorhamnetin-3-glucoside	2.80	477.10646	C_22_H_22_O_12_	315.0695 (38)	+	−
9.	Apigenin-7-*O*-glucoside	2.88	431.109907	C_21_H_20_O_10_	269.0449 (100)	+	−
10.	Hesperidin	2.92	609.18546	C_28_H_34_O_15_	301.0695 (100)	+	+
11.	Hispidulin rutinoside	2.95	607.17094	C_28_H_32_O_15_	301.0699 (100), 299.0559 (24)	+	+
12.	Hispidulin-7-*O*-glucoside	3.03	461.11113	C_22_H_22_O_11_	283.0234 (13), 299.0561(8)	+	−
13.	6-Hydroxyluteolin-7-*O*-glucoside	3.10	463.08011	C_21_H_20_O_12_	301.0350 (100)	+	−
14.	Luteolin	3.11	285.03995	C_15_H_10_O_6_	133.0284 (12), 151.0029 (12), 175.0395 (9),199.0395 (8)	−	+
15.	Luteolin-7-*O*-glucuronide	3.11	461.07495	C_21_H_18_O_12_	285.0385 (100)	+	+
16.	Isorhamnetin	3.20	315.05001	C_16_H_12_O_7_	300.0255 (100), 301.0311 (39)	+	+
17.	Luteolin 3’-acetyl-*O*-glucuronide isomer I	3.29	503.08570	C_23_H_20_O_13_	285.0381 (100), 443.0587 (100), 381.0606 (35), 399.0720 (28)	+	+
18.	Luteolin 3’-acetyl-*O*-glucuronide isomer II	3.38	503.08550	C_23_H_20_O_13_	285.0366 (100)	+	+
19.	Apigenin	3.49	269.04559	C_15_H_10_O_5_	117.0356 (12), 149.0356 (8), 225.0560 (5)	−	+
20.	Hesperetin	3.58	301.07074	C_16_H_14_O_6_	242.0571 (87), 284.286.0468 (55), 164.0108(54), 151.0036 (35)	−	+
21.	Diosmetin	3.58	299.05595	C_16_H_12_O_6_	284.0310 (100)	−	+
22.	Luteolin 3’-acetyl-*O*-glucuronide	3.62	503.08594	C_23_H_20_O_13_	285.4652 (100), 443.0598 (76)	+	+
23.	Rosmanol isomer	4.07	345.16874	C_20_H_26_O_5_	301.1782 (100), 283.1673 (68), 284.1719 (29)	−	+
24.	Pectolinarigenin	4.15	313.07285	C_17_H_14_O_6_	298.0464 (100), 283.0235 (52), 255.0285 (17), 163.0034 (10), 227.0344 (6), 117.0350 (4)	+	+
25.	Rosmanol	4.30	345.17145	C_20_H_26_O_5_	301.1782 (100), 283.1673 (65), 284.1719 (32)	+	+
26.	Genkwanin	4.58	283.06224	C_16_H_12_O_5_	268.0381 (100), 240.0431 (6)	+	+
27.	Rosmanol isomer	4.60	345.17190	C_20_H_26_O_5_	284.1704 (100)	−	+
28.	Rosmadial isomer	4.98	343.15577	C_20_H_24_O_5_	299.1618 (55), 243.1010 (9)	+	+
29.	Rosmanol methyl ether	5.08	359.14801	C_21_H_18_O_5_	315.1577 (19)	−	+
30.	Rosmanol	5.15	345.16890	C_20_H_26_O_5_	283.1669 (69)	−	+
31.	Carnosol isomer	5.49	329.17480	C_20_H_26_O_4_	285.1825 (100)	−	+
32.	Rosmadial	5.61	343.15305	C_20_H_24_O_5_	299.1623 (100)	+	+
33.	Trihydroxy-methoxyflavone	5.70	299.16397	C_16_H_12_O_6_	284.0310 (100)	−	+
34.	Carnosol	5.75	329.17666	C_20_H_26_O_4_	285.1834 (100)	+	+
35.	Carnosic acid	5.76	331.18358	C_20_H_28_O_4_	287.1649 (100)	+	+
36.	Rosmaridiphenol	6.16	315.19780	C_20_H_26_O3	285.1843 (19)	+	+
37.	Rosmadial isomer	6.20	343.15249	C_20_H_24_O_5_	299.1598 (56)	+	+
38.	Rosmadial isomer	6.56	343.15233	C_20_H_24_O_5_	299.1602 (68)	+	+
39.	12-methoxy-carnosic acid	6.99	345.20823	C_21_H_30_O_4_	301.2157 (100), 286.1923 (65)	+	+
40.	Betulinic acid	8.05	455.34934	C_30_H_48_O_3_	−	+	−
41.	Ursolic acid	8.10	455.35307	C_30_H_48_O_3_	−	+	+

T_1_: Fresh tissue homogenization; T_3_: Fresh homogenized tissue extract fermentation; * Fragmentation values are followed by their intensity % in parenthesis.

**Table 3:** Analysis of bioactive compounds in fresh leaf (T_2_) and dry leaf decoction (T_5_) and their respective fermented extracts (T_4_ and T_6_) of *R. officinalis* by UHPLC-ESI-QTOF-MS.

Sl No	Compound	RT (min)	Mass [M − H]^−^ (*m/z*)	Formula	Fragments	T_2_	T_4_	T_5_	T_6_
1.	Quinic acid	0.35	191.05670	C_7_H_12_O_6_	85.0301 (39), 93.0354 (18), 127.0406 (15)	+	+	+	+
2.	Syringic acid	0.42	197.04643	C_9_H_10_O_5_	135.0450 (100), 123.0450 (100), 72.9947 (84), 179.0349 (54)	+	+	+	+
3.	Chlorogenic acid	0.67	353.08621	C_16_H_18_O_9_	191.0560 (28)	+	−	+	−
4.	Caffeic acid	0.78	179.03600	C_9_H_8_O_4_	135.0444 (100), 134.0372 (19)	+	+	+	+
5.	4-*O*-Caffeoyl quinic acid	1.00	353.08940	C_16_H_18_O_9_	173.0439 (100), 179.0329 (37), 135.0434 (14)	+	−	+	−
6.	*p*-Coumaric acid	1.30	163.04015	C_9_H_8_O_3_	119.0509 (100)	+	+	+	+
7.	Gallocatechin	1.40	305.07127	C_15_H_14_O_7_	225.1123 (49), 96.9595 (24)	+	+	+	+
8.	6-Hydroxyluteolin-7-*O*-glucoside	1.89	463.08849	C_21_H_20_O_12_	286.0427 (100), 301.0350 (69), 285.7613 (44)	+	−	+	−
9.	Luteolin-7-*O*-glucoside	2.18	447.09508	C_21_H_20_O_11_	285.0413 (53)	+	−	+	+
10.	Luteolin 7-*O*-rutinoside	2.18	593.15454	C_27_H_30_O_15_	285.0431 (11)	+	+	+	+
11.	Scutellarin	2.21	461.07517	C_21_H_18_O_12_	285.0405 (100), 113.0252 (9), 175.0252 (6)	−	−	−	+
12.	Rosmarinic acid-3-*O*-glucoside	2.23	521.13273	C_24_H_26_O_13_	359.0792 (100), 324.0832 (78), 323.0785 (60)	+	+	+	+
13.	Salvianolic acid B	2.29	717.15054	C_36_H_30_O_16_	519.0891 (100), 339.0500 (15)	+	+	+	+
14.	Isorhamnetin-3-*O*-glucoside	2.33	477.10584	C_22_H_22_O_12_	315.0539(38)	+	+	+	+
15.	Sagerinic acid	2.52	719.16630	C_36_H_32_O_16_	359.0761 (100), 179.0336 (20), 161.0223 (16)	+	+	+	+
16.	Rosmarinic acid	2.56	359.07835	C_18_H_16_O_8_	161.0238 (100), 197.0447 (64), 179.0341 (57), 133.0290 (35), 72.9940 (6)	+	+	+	+
17.	Apigenin-7-*O*-glucoside	2.59	431.09779	C_21_H_20_O_10_	269.0420 (100), 149.0969 (3)	−	−	+	−
18.	Hesperidin	2.63	609.18493	C_28_H_34_O_15_	301.0714 (100)	+	+	+	+
19.	Salvianolic acid A	2.64	493.11382	C_26_H_22_O_10_	295.0615 (100), 185.0224 (43), 109.0289 (11)	+	+	+	+
20.	Diosmin	2.66	607.17017	C_28_H_32_O_15_	301.0704 (100), 299.0551 (59)	+	+	+	+
21.	Hispidulin-7-*O*-glucoside	2.68	461.11080	C_22_H_22_O_11_	283.0235 (12), 299.0552 (9)	+	−	+	−
22.	Luteolin-7-*O*-glucuronide	2.79	461.07517	C_21_H_18_O_12_	286.0430 (100), 285.0399 (38)	+	+	+	+
23.	Hesperetin	2.87	301.07245	C_16_H_14_O_6_	286.0459 (12), 164.0100 (4)	−	−	+	−
24.	Methyl rosmarinate	2.99	373.09453	C_19_H_18_O_8_	175.0403 (100), 357.0610 (61), 198.0477 (33), 179.0367 (22), 135.0465 (11)	+	+	+	+
25.	Luteolin 3’-acetyl-*O*-glucuronide isomer I	3.10	503.08463	C_23_H_20_O_1_	399.0721 (100), 285.7547 (6)	+	+	+	+
26.	Luteolin	3.11	285.04114	C_15_H_10_O_6_	133.0302 (18), 151.0051 (6), 175.0410 (5), 199.0414 (4)	+	+	+	+
27.	Isorhamnetin	3.17	315.05133	C_16_H_12_O_7_	300.0279 (100), 301.0332 (32)	+	+	+	+
28.	Luteolin 3’-acetyl-*O*-glucuronide isomer II	3.27	503.08550	C_23_H_20_O_1_	286.0415 (100), 285.7547 (62), 443.0607 (60), 399.0721 (7)	+	+	+	+
29.	Apigenin	3.47	269.04675	C_15_H_10_O_5_	117.0350 (8), 151.0043(7), 225.0576 (4)	+	+	+	+
30.	Luteolin 3’-acetyl-*O*-glucuronide	3.62	503.08530	C_23_H_20_O_13_	286.0415 (100), 443.0607 (47), 285.7547 (38)	+	+	+	+
31.	Diosmetin	3.64	299.05647	C1_6_H_12_O_6_	284.0339 (100)	+	+	+	+
32.	Rosmanol isomer	4.07	345.17252	C_20_H_26_O_5_	301.1802 (100), 283.1698 (67)	+	+	+	+
33.	3,7 Dihydroxy-dimethoxyflavone	4.15	313.07320	C_17_H_14_O_6_	298.0473 (100), 283.0243 (70), 255.0306 (19), 269.0464 (12)	−	−	+	−
34.	Pectolinarigenin	4.15	313.07258	C_17_H_14_O_6_	298.0469 (100), 283.0240 (63)	+	+	−	+
35.	Rosmanol	4.30	345.17100	C_20_H_26_O_5_	283.8834 (18)	+	+	+	+
36.	Pectolinarigenin isomer	4.37	313.07211	C_17_H_14_O_6_	298.0471 (91), 283.0233 (63), 255.0290 (24)	+	−	−	−
37.	Rosmanol isomer	4.57	345.17200	C_20_H_26_O_5_	284.1750 (13), 283.1706 (11)	+	+	+	+
38.	Genkwanin	4.58	283.06218	C1_6_H_12_O_5_	268.0398 (79), 240.0434 (5)	+	+	+	+
39.	Rosmadial isomer	4.80	343.15636	C_20_H_24_O_5_	299.1669 (32)	+	+	+	+
40.	Rosmanol isomer	5.17	345.17210	C_20_H_26_O_5_	283.8769 (39)	+	+	+	+
41.	Rosmanol methyl ether	5.07	359.18598	C_21_H_28_O_5_	283.1703 (100), 300.1747 (82)	+	+	&	&
42.	Asiatic acid	5.57	487.34312	C_30_H_48_O_5_	&	+	&	&	&
43.	Rosmadial	5.69	343.15590	C_20_H_24_O_5_	299.1645 (9)	+	+	+	+
44.	Trihydroxy- methoxyflavone	5.69	299.16340	C1_6_H_12_O_6_	284.0333 (100)	&	+	&	&
45.	Carnosol	5.75	329.17690	C_20_H_26_O_4_	286.1870 (100), 285.1845 (92)	+	+	+	+
46.	Carnosic acid	5.76	331.19253	C_20_H_28_O_4_	287.2007 (100)	+	+	+	+
47.	Rosmadial isomer	6.04	343.17192	C_20_H_24_O_5_	299.1653 (15)	-	+	&	+
48.	Rosmaridiphenol	6.16	315.19689	C_20_H_28_O_3_	284.1860 (4)	+	+	+	+
49.	Carnosol isomer	6.17	329.17560	C_20_H_26_O_4_	286.1880 (100), 285.1852 (58)	+	&	+	&
50.	12-methoxy-carnosic acid	6.99	345.20853	C_21_H_30_O_4_	286.1943 (100), 301.2186 (81)	+	+	+	+
51.	Micromeric acid	7.64	453.33442	C_30_H_46_O_3_	&	+	&	+	&
52.	Betulinic acid	8.05	455.34921	C_30_H_48_O_3_	&	+	&	+	&
53.	Ursolic acid	8.10	455.35205	C_30_H_48_O_3_	&	+	+	+	+

T_2_: Fresh leaf decoction; T_4_: Fresh leaf decoction fermentation; T_5_: Dry leaf decoction; T_6_: Dry leaf decoction fermentation.

**Table 4:** Analysis of bioactive constituents by UHPLC-ESI-QTOF-MS in Soxhlet extract (T_7_) and sonicated extracts (water and methanol—T_8_ and T_9_) of *R. officinalis.*

Sl No	Compound	RT (min)	Mass [M − H]^−^ (*m/z*)	Formula	Fragments	T_7_	T_8_	T_9_
1.	Quinic acid	0.35	191.05628	C_7_H_12_O_6_	85.0299 (34), 93.0353 (17), 127.0403 (12)	+	+	+
2.	Syringic acid	0.42	197.04569	C_9_H_10_O_5_	135.0450 (100), 123.0450 (75), 72.9947 (60), 179.0349 (54)	+	-	+
3.	Chlorogenic acid	0.56	353.08506	C_16_H_18_O_9_	191.0545 (26)	&	&	+
4.	Caffeic acid	0.82	179.03589	C_9_H_8_O_4_	135.0441 (100), 134.0370 (23)	+	&	+
5.	4-*O*-Caffeoyl quinic acid	0.85	353.08949	C_16_H_18_O_9_	173.0437 (100), 191.0544 (27), 179.0334 (6)	&	&	+
6.	*p*-Coumaric acid	1.28	163.04014	C_9_H_8_O_3_	119.0509 (100)	+	+	+
7.	Gallocatechin	1.81	305.06932	C_15_H_14_O_7_	96.9588 (65), 225.1109 (59)	+	+	+
8.	6-Hydroxyluteolin-7-*O*-glucoside	1.97	463.08518	C_21_H_20_O_12_	301.0350 (69), 285.7613 (44)	&	&	+
9.	3-*p*-coumaroylquinic acid	2.1	337.10122	C_16_H_18_O_8_	163.0397 (100), 119.0506 (32)	+	&	&
10.	Luteolin-7-*O*-glucoside	2.23	447.09286	C_21_H_20_O_11_	285.0377 (29)	+	+	+
11.	Luteolin 7-*O*-rutinoside	2.24	593.15126	C_27_H_30_O_15_	285.0380 (2)	+	&	+
12.	Rosmarinic acid-3-*O*-glucoside	2.25	521.12957	C_24_H_26_O_13_	359.0740 (100), 323.0737 (89), 179.0337 (15)	+	&	+
13.	Apigenin-7-*O*-glucunoride	2.51	445.07602	C_21_H_18_O_11_	269.0437 (100), 113.0255 (10), 175.0252 (9)	&	+	&
14.	Sagerinic acid	2.52	719.15575	C_36_H_32_O_16_	359.0761 (100), 179 (50), 161.0223 (17)	+	&	+
15.	Rosmarinic acid	2.56	359.07651	C_18_H_16_O_8_	161.0229 (100), 197.0434 (78), 179.0330 (60), 133.0285 (15), 72.9934 (8)	+	+	+
16.	Isorhamnetin-3-*O*-glucoside	2.56	477.10320	C_22_H_22_O_12_	315.0466 (32), 300.0246 (5)	+	&	+
17.	Apigenin-7-*O*-glucoside	2.59	431.09762	C_21_H_20_O_10_	269.0435 (100)	+	+	+
18.	Isoferulic acid	2.62	193.05102	C_10_H_10_O_4_	134.0386 (89), 133.0295 (75), 178.0271 (12)	+	&	&
19.	Isorhamnetin-3-*O*-rutinoside	2.63	623.15851	C_28_H_32_O_16_	315.0471 (9)	+	&	+
20.	Hispidulin-7-*O*-glucuronide	2.64	475.08642	C_22_H_20_O_12_	299.0543 (100), 285.0367 (50), 283.0313 (3)	&	+	&
21.	Hesperidin	2.64	609.18172	C_28_H_34_O_15_	301.0674 (100)	+	&	+
22.	Salvianolic acid A	2.64	493.11132	C_26_H_22_O_10_	295.0615 (100), 185.0224 (43), 109.0289 (11)	+	&	&
23.	Diosmin	2.66	607.16666	C_28_H_32_O_15_	299.0527 (57), 284.0309 (4)	+	+	+
24.	Hispidulin-7-*O*-glucoside	2.69	461.11138	C_22_H_22_O_11_	298.0477 (18), 283.0234 (7)	+	&	+
25.	Kaempferol-7-*O*-hexoside	2.72	447.09094	C_21_H_20_O_11_	285.0382 (100)	+	&	+
26.	Luteolin-7-*O*-glucuronide	2.83	461.06888	C_21_H_18_O_12_	285.7541 (100)	+	+	+
27.	Phlorizin	2.95	435.13210	C_21_H_24_O_10_	273.0767 (100), 167.0349 (28), 125.0247 (5)	+	&	&
28.	Luteolin	3.12	285.04024	C_15_H_10_O_6_	133.0300 (14), 151.0042 (10), 175.0409 (8), 199.0406 (6)	+	+	+
29.	Isorhamnetin	3.21	315.04996	C_16_H_12_O_7_	300.0248 (100), 301.0286 (41)	+	&	+
30.	Luteolin 3’-acetyl-*O*-glucuronide isomer I	3.29	503.08148	C_23_H_20_O_13_	399.0707(100), 285.4652 (9), 443.0598 (5)	&	+	&
31.	Methyl rosmarinate	3.30	373.08946	C_19_H_18_O_8_	135.0441 (100), 175.0397 (100), 179.0346 (85), 197.0449 (79)	+	&	+
32.	Luteolin 3’-acetyl-*O*-glucuronide II	3.38	503.08230	C_23_H_20_O_13_	286.0407 (100), 285.4649 (38)	+	+	+
33.	Apigenin	3.50	269.04386	C_15_H_10_O_5_	117.0347 (11), 151.0036 (11), 225.0557 (5)	+	+	+
34.	Salvianolic acid B	2.29	717.14025	C_36_H_30_O_16_	519.0912 (57), 339.0471 (37)	+	&	&
35.	Hesperetin	3.58	301.07242	C_16_H_14_O_6_	164.0114 (11), 286.0827 (5)	&	+	&
36.	Luteolin 3’-acetyl-*O*-glucuronide	3.62	503.08259	C_23_H_20_O_13_	443.0607 (100), 285.7547 (68)	+	+	+
37.	Diosmetin	3.64	299.05429	C_16_H_12_O_6_	284.0298 (100)	+	+	+
38.	Rosmanol isomer	4.07	345.16839	C_20_H_26_O_5_	301.1773 (100), 283.1668 (65)	+	&	+
39.	Pectolinarigenin	4.15	313.06990	C_17_H_14_O_6_	298.0474 (100), 283.0237 (59), 255.0295 (24), 163.0037 (15), 117.0345 (7)	+	+	+
40.	Rosmanol	4.30	345.16852	C_20_H_26_O_5_	301.1773 (100), 283.1668 (57)	+	&	+
41.	Pectolinarigenin isomer	4.37	313.06892	C_17_H_14_O_6_	298.0471 (91), 283.0233 (63), 255.0290 (24)	+	&	&
42.	Triptolidenol	4.45	375.15421	C_20_H_24_O_7_	331.1526 (13), 244.1082 (9), 313.1430 (7)	&	&	+
43.	Rosmadial isomer	4.54	343.15249	C_20_H_24_O_5_	299.1610 (9)	+	&	+
44.	Genkwanin	4.58	283.06017	C_16_H_12_O_5_	268.0379 (89), 117.0353 (5), 151.0039 (4)	+	+	+
45.	Rosmanol isomer	4.59	345.16789	C_20_H_26_O_5_	284.1687 (40), 283.8801 (23)	+	&	+
46.	Rosmanol isomer	4.80	345.16793	C_20_H_26_O_5_	283.1668 (19)	+	&	+
47.	Asiatic acid	5.47	487.34312	C_30_H_48_O_5_	&	+	&	&
48.	Rosmadial	5.71	343.15314	C_20_H_24_O_5_	299.1616 (10)	+	+	+
49.	Rosmanol isomer	5.71	345.16811	C_20_H_26_O_5_	283.1670 (12)	&	+	&
50.	Carnosol	5.75	329.17532	C_20_H_26_O_4_	285.1833 (100)	+	+	+
51.	Epirosmanol methyl ether	5.85	359.18381	C_21_H_28_O_5_	283.1665 (97), 329.1719 (16), 300.1713 (15)	+	&	+
52.	Rosmadial isomer	6.05	343.15233	C_20_H_24_O_5_	299.1621 (11)	+	&	+
53.	Carnosic acid	6.58	331.19123	C_20_H_28_O_4_	287.1982 (100)	+	&	+
54.	Corosolic acid	6.61	471.34212	C_30_H_48_O_4_	&	&	&	+
55.	12-methoxy-carnosic acid	6.99	345.20630	C_21_H_30_O_4_	301.2170 (100), 287.1938 (64)	+	&	+
56.	Micromeric acid	7.84	453.34261	C_30_H_46_O_3_	&	+	&	+
57.	Betulinic acid	8.05	455.34934	C_30_H_48_O_3_	&	+	&	+
58.	Ursolic acid	8.10	455.35011	C_30_H_48_O_3_	&	+	+	+

T_7_: Soxhlet extraction; T_8_: Sonic extraction—aqueous; T_9_: Sonic extraction—methanol.

**Table 5:** Results of analysis of calibration curve and limits of quantification.

	Standard	Purity (%)	Formula	Molecular Weight	LOQ (ng/mL)	Calibration Range (ng/mL)	Calibration Equations	Slope (R^2^)
1	Caffeic acid	98.0	C_9_H_8_O_4_	180.00	6.0	6–250	*y* = 0.00523*x* + 0.00157	0.9993
2	Rosmarinic acid	98.0	C_18_H_16_O_8_	360.31	6.0	6–250	*y* = 0.00374*x* − 0.00269	0.9998
3	Luteolin-7-*O*-glucoside	98.0	C_21_H_20_O_11_	448.38	6.0	6–250	*y* = 0.00347*x* − 0.00156	0.9994
4	Carnosol	100.0	C_20_H_26_O_4_	330.40	6.0	6–250	*y* = 0.00673*x* + 0.03215	0.9982
5	Carnosic acid	96.0	C_20_H_28_O_4_	332.43	24.0	24–1000	*y* = 9.06876 × 10^−5^*x* + 0.00303	0.9984
6	Ursolic acid	97.0	C_30_H_48_O_3_	456.70	24.0	24–1000	*y* = 0.00147*x* − 0.03017	0.9938

Limits of quantification (LOQ).

## Supplementary Materials

Figure S1: QTOF-MS spectrum of caffeic acid, Figure S2: QTOF-MS spectrum of rosmarinic acid, Figure S3: QTOF-MS spectrum of luteolin-7-*O*-glucoside, Figure S4: QTOF-MS spectrum of carnosol, Figure S5: QTOF-MS spectrum of carnosic acid, Figure S6: QTOF-MS spectrum of ursolic acid.
