# Supplementary material for: Full-Spectrum Analysis of Bioactive Compounds in Rosemary (Rosmarinus officinalis L.) as Influenced by Different Extraction Methods
Source: Molecules. 2020 Oct 9;25(20):4599. doi: 10.3390/molecules25204599 (PMC7587196; doi:10.3390/molecules25204599)
Supplement: Supplementary file 1 [file molecules-25-04599-s001.pdf]

QTOF-MS spectrum of reference standards- caffeic acid, rosmarinic acid, luteolin-7-*O*-glucoside, carnosol, carnosic acid and ursolic acid

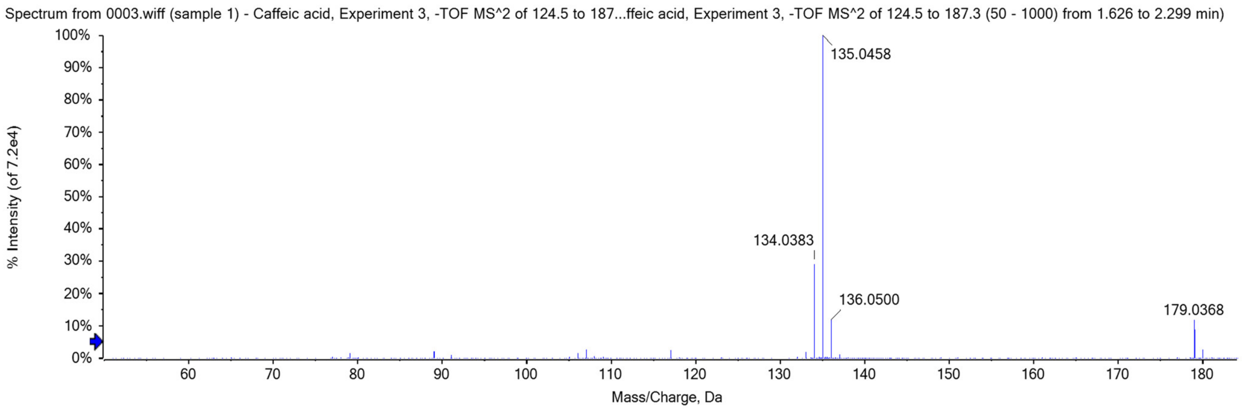

Figure S1. QTOF-MS spectrum of caffeic acid.

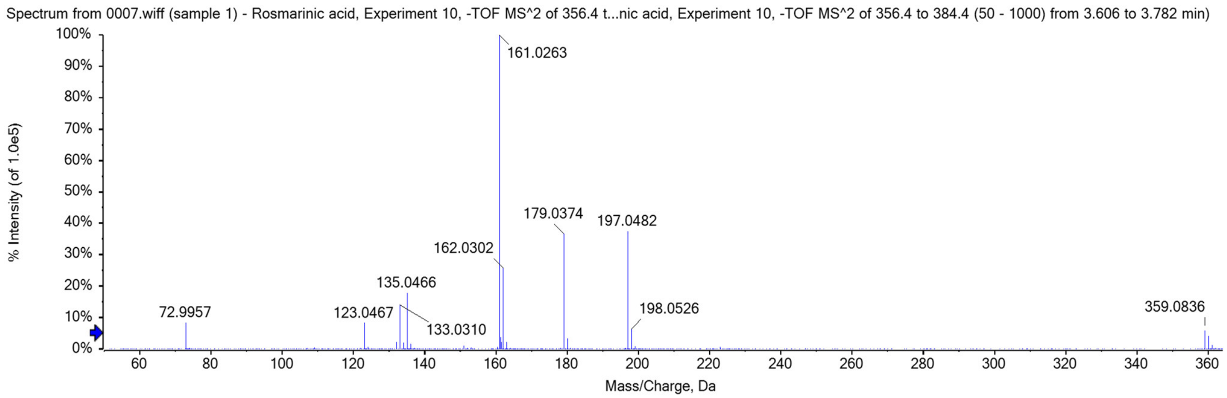

Figure S2. QTOF-MS spectrum of rosmarinic acid.

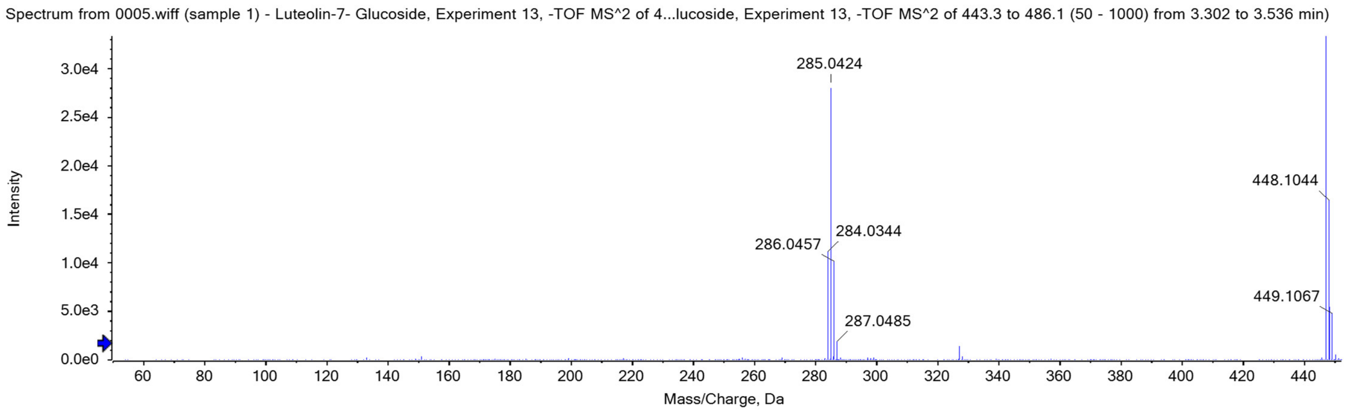

Figure S3. QTOF-MS spectrum of luteolin-7-*O*-glucoside.

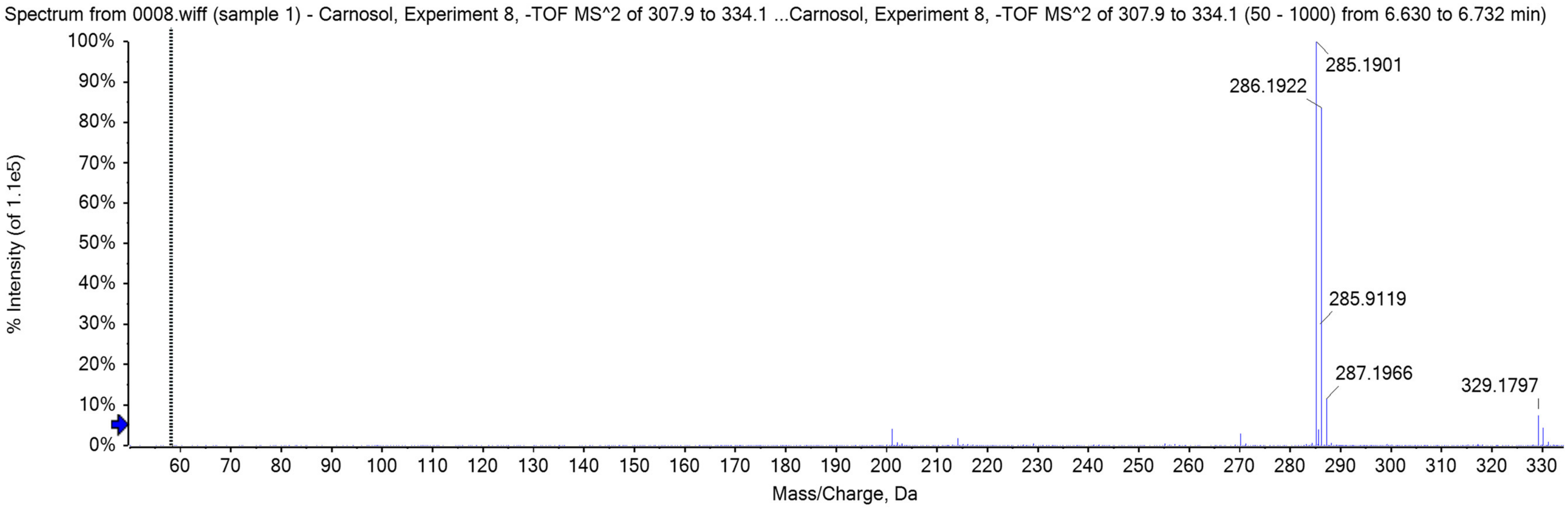

Figure S4. QTOF-MS spectrum of carnosol.

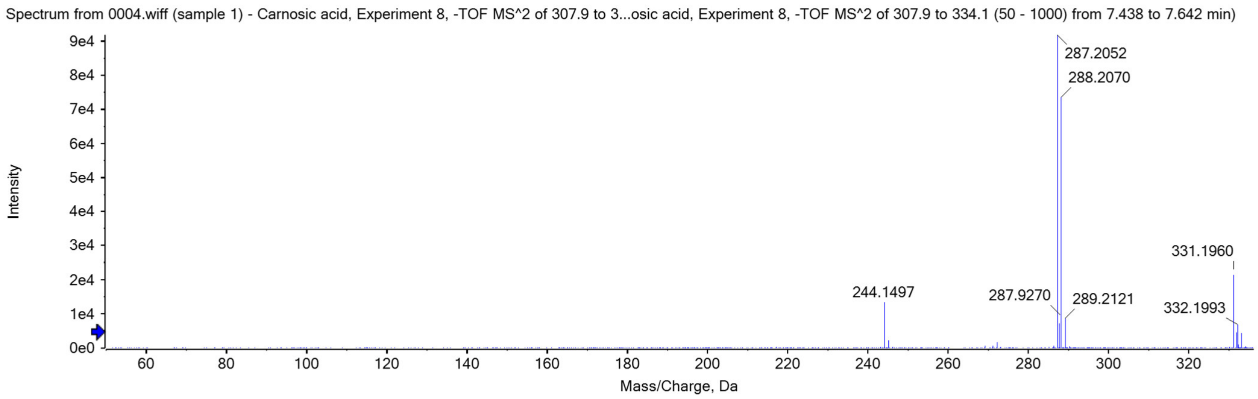

Figure S5. QTOF-MS spectrum of carnosic acid.

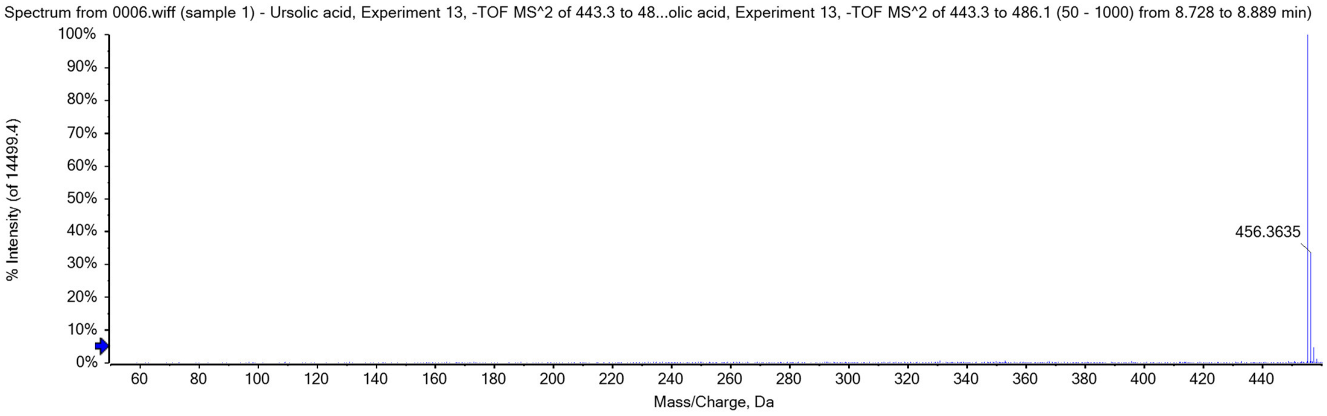

Figure S6. QTOF-MS spectrum of ursolic acid.
